# Host-Imposed Copper Poisoning Impacts Fungal Micronutrient Acquisition during Systemic *Candida albicans* Infections

**DOI:** 10.1371/journal.pone.0158683

**Published:** 2016-06-30

**Authors:** Joanna Mackie, Edina K. Szabo, Dagmar S. Urgast, Elizabeth R. Ballou, Delma S. Childers, Donna M. MacCallum, Joerg Feldmann, Alistair J. P. Brown

**Affiliations:** 1 Aberdeen Fungal Group, School of Medicine, Medical Sciences & Nutrition, Institute of Medical Sciences, University of Aberdeen, Foresterhill, Aberdeen, AB25 2ZD, United Kingdom; 2 Trace Element Speciation Laboratory, Department of Chemistry, College of Physical Science, University of Aberdeen, Meston Walk, Aberdeen AB24 3UE, United Kingdom; Louisiana State University, UNITED STATES

## Abstract

Nutritional immunity is a process whereby an infected host manipulates essential micronutrients to defend against an invading pathogen. We reveal a dynamic aspect of nutritional immunity during infection that involves copper assimilation. Using a combination of laser ablation inductively coupled mass spectrometry (LA-ICP MS) and metal mapping, immunohistochemistry, and gene expression profiling from infected tissues, we show that readjustments in hepatic, splenic and renal copper homeostasis accompany disseminated *Candida albicans* infections in the mouse model. Localized host-imposed copper poisoning manifests itself as a transient increase in copper early in the kidney infection. Changes in renal copper are detected by the fungus, as revealed by gene expression profiling and fungal virulence studies. The fungus responds by differentially regulating the Crp1 copper efflux pump (higher expression during early infection and down-regulation late in infection) and the Ctr1 copper importer (lower expression during early infection, and subsequent up-regulation late in infection) to maintain copper homeostasis during disease progression. Both Crp1 and Ctr1 are required for full fungal virulence. Importantly, copper homeostasis influences other virulence traits—metabolic flexibility and oxidative stress resistance. Our study highlights the importance of copper homeostasis for host defence and fungal virulence during systemic disease.

## Introduction

Micronutrients such as ferrous or cuprous ions are scarce, yet essential for life. They play central roles in many biological processes, executing both structural and catalytic functions [1]. However, these ions are toxic in excess. For example, they promote free radical generation via the Fenton reaction, which causes lipid and protein peroxidation [2]. Therefore, metal partitioning and distribution must be strictly controlled to avoid cellular damage. Metal availability resides at the centre of pathogen-host interactions. In a process termed ‘nutritional immunity’, the host manipulates the availability of specific micronutrients to the disadvantage of invading microbes, either starving them for metal ions [3,4], or poisoning them with metal overload [5–7]. In response, pathogenic microbes activate specific mechanisms to overcome this metal deprivation or excess [4,8,9].

Recently, we explored the dynamics of global iron homeostasis in the host during systemic infections caused by the opportunistic fungal pathogen *Candida albicans*. This fungus is a commensal microbe that colonises the gut and mucosal surfaces of healthy individuals. In immunocompromised patients, it causes life-threatening infections characterised by extremely high mortality rates (40%) [10,11]. We showed previously that the iron landscape of the kidney is profoundly affected by systemic fungal infection [12]. During disseminated candidiasis, the liver synthesizes increased amounts of hepcidin, which inhibits iron release from tissue stores. Also, the recycling of erythrocytes (red blood cells) is perturbed in splenic macrophages of the reticuloendothelial system, requiring the kidney to become involved in erythrocyte recycling. Consequently, iron concentrations increase in the renal medulla. Meanwhile, through the action of haem oxygenases, immune infiltrates in the kidney prevent this iron from reaching the fungal cells. This generates zones of iron starvation around the fungal lesions, representing a classical nutritional immunity mechanism. The fungus counteracts these changes by switching its iron acquisition strategies from *FTR1*-dependent reductive iron acquisition to *HMX1*-dependent haem iron acquisition [12].

The acquisition, partitioning and mobilisation of cellular iron depend on copper availability, and this dependency is evolutionarily conserved [13]. In mammalian cells, copper-containing oxidases such as hephaestin and ceruloplasmin mediate Fe^3+^ loading onto the major blood iron carrier, transferrin, for transport to distant tissues [13,14]. Meanwhile, reductases enable the import of iron via the DMT1 transporter, and its subsequent intracellular storage in ferritin [13]. Fungi also utilise copper ferroxidases to acquire iron from the environment, via the reductive iron acquisition pathway (e.g. Fet3 in *C*. *albicans* [15], and FetC in *Aspergillus fumigatus* [16]). Unlike intracellular iron redistribution, which involves non-specific metal trafficking between intracellular locations, copper ions are relayed via specific metallochaperones to their cognate protein targets [13,17]. For example, murine ATOX1 relays copper to the trans-Golgi copper transporting ATPase ATP7B [18], CCS to superoxide dismutase SOD1 [19], and COX17 to mitochondrial cytochrome c oxidase [20]. In contrast to the relatively well-researched area of iron nutritional immunity, there have been few studies of the copper nutritional immunity mechanisms that operate during microbial infections. Notable examples include copper poisoning of macrophage-engulfed *Salmonella* Typhimurium [21] and *Mycobacterium tuberculosis* cells [5], and the adaptation of *Cryptococcus neoformans* to changes in copper levels during systemic infection [6,22].

Here we explore the importance of copper nutritional immunity during the development of *C*. *albicans* infections in mice. We show that systemic candidiasis triggers adjustments in copper uptake and release by organs that are peripheral to the major site of infection in the kidney, such as the liver and spleen. Intriguingly, these adjustments coincide with infection-associated shifts in iron metabolism in these organs. Renal copper levels increase during the early stages of fungal colonisation, and decrease late in infection. *C*. *albicans* counteracts copper excess early in infection by high-level expression of the Crp1 copper efflux pump. As the infection progresses, Crp1 expression is down-regulated and Ctr1 high affinity copper importer is up-regulated. Both the efflux pump and importer are required for full fungal virulence in the mouse model, revealing the importance of dynamic host-fungal interactions during nutrient immunity. We conclude that the maintenance of cellular copper homeostasis during periods of copper excess and subsequent starvation is essential for *C*. *albicans* virulence.

## Materials and Methods

### *Candida albicans* strains and growth conditions

Experiments were performed with *C*. *albicans* clinical isolate SC5314 [23] or mutants constructed in this background (below). Unless specified otherwise, cells were grown at 30°C in YPD medium [24] or YNB (0.67% yeast nitrogen base without amino acids) medium, supplemented with 2% appropriate carbon sources, or with stressor at the specified concentration.

*C*. *albicans* mutants were constructed using the *Clox* system with nourseothricin selection [25]. To create the homozygous *ctr1*/*ctr1* deletion mutant, the *NAT1-Clox* cassette was PCR amplified using primer pairs CTR1_CLOx_F_JP plus CTR1_CLOx_R_JP, and CTR1_CLOx_F3_JP plus CTR1_CLOx_R3_JP (S1 Table) and used to transform SC5314 to sequentially delete both *CTR1* alleles. To construct the *ctr1*/*CTR1* reintegrant strain, the wild type *CTR1* sequence was reintroduced into its native chromosomal locus. The *CTR1* locus was PCR amplified using primers SpeI_F_CTR1_ReINT_JP and Not1_R_CTR1_ReINT_JP (S1 Table), digested with *Spe*I and *Not*I, and cloned into pLNMCL [25]. The plasmid was then digested with *Bst*API and used to transform the *ctr1/ctr1* deletion mutant selecting for nourseothricin resistance.

To create the homozygous *crp1*/*crp1* null mutant, the *NAT1-Clox* cassette was PCR amplified using primer pairs CRP1_CLOx_F_JP plus CRP1_CLOx_R_JP, and CRP1_CLOX_F2_JP plus CRP1_CLOX_R2_JP (S1 Table), with both *CRP1* alleles deleted sequentially in *C*. *albicans* SC5314. To construct the *crp1/CRP1* reintegrant strain, the wild type *CRP1* locus was PCR amplified using primers Not1_F_CRP1_ReINT_JP plus Not1_R_CRP1_ReINT_JP (S1 Table), digested with *Not*I and cloned into pLNMCL. This plasmid was then digested with *Kpn*2I, and used to transform the *crp1/crp1* null mutant.

The genotype of each mutant and the ploidy of the target locus were verified by qRT-PCR with Roche LightCycler 480 system with specific primer pairs (S2 Table).

### Animal experiments

Animal experiments were performed as described previously [12]. Briefly, *C*. *albicans* strains were pre-grown overnight at 30°C in NGY broth [26]. *C*. *albicans* inocula (10^4^−10^5^ CFU/g body mass) were injected into the lateral tail veins of 6–10 week old specific pathogen-free female BALB/c mice (Harlan, UK). For the *CTR1* virulence study, the infection doses (CFU/g animal weight x 10^4^) were as follows: SC5314, 1.9 ± 0.1; *ctr1* deletion mutant, 1.1 ± 0.1; *ctr1/CTR1* reintegrant, 4.2 ± 0.2. For the *CRP1* virulence study, the infection doses (CFU/g animal weight x 10^4^) were: SC5314, 3.2 ± 0.1; *crp1* deletion mutant, 1.0 ± 0.03; *crp1/CRP1* reintegrant, 2.5 ± 0.1. Infections were allowed to proceed for 3 days, with six animals randomly assigned to every experimental group (the group size was determined by Power calculations), and fungal loads determined in kidneys [27]. Early infections were analysed after 24 h and advanced infections after 96 h. Tissues were placed on dry ice and stored at -80°C before processing for histology or ICP-MS, as described [12]. Periodic acid and Schiff staining [28] was used to assess the histology of fresh-frozen tissues. Alternatively, for transcript profiling, the harvested organs were stored in RNAlater (Qiagen, Crawley, UK) [12].

### *Ex vivo* renal epithelium model

The *ex vivo* experiments were performed as described elsewhere [29]. Briefly, *C*. *albicans* strains were grown overnight at 30°C in YPD, washed three times in PBS, and co-incubated (MOI = 1) with M-1 mouse kidney cortical collecting duct epithelial cells (CRL-2038, ATCC) for up to 24 h in DMEM:Ham’s F12 supplemented with 2 mM glutamine (Gibco Life Technologies), 5 μM dexamethasone (Hspira UK Limited), 5% foetal bovine serum with 5% CO_2_ at 37°C. KC production was measured by ELISA [29]. Renal epithelial cell damage was assessed by assaying lactate dehydrogenase release into the culture supernatant [29]. For histology, the cells were fixed in 4% formaldehyde for 12 h at 8°C, washed three times with PBS and stained using Periodic acid and Schiff [28]. Mann-Whitney U test with two-tailed t-test was used to assess statistical differences between the two groups.

### Immunohistochemistry

Specific antigens in kidney, liver and spleen sections were detected using the Vectastain Elite ABC System (VectorLabs, Orton Southgate, UK), according to manufacturer’s instructions. The following primary mouse-specific antibodies were used: ATP7A, rabbit, polyclonal (Abcam, Cambridge, UK); ATP7B, rabbit, polyclonal to ATP7B C-terminal region (Abcam, Cambridge, UK); ceruloplasmin, rabbit, polyclonal (Abcam, Cambridge, UK); CTR1, rabbit, polyclonal to SLC31A1/CTR1 (Abcam, Cambridge, UK); F4/80, rat, monoclonal (AbDSerotec, Kidlington, UK); HO-1, rabbit, polyclonal (Abcam, Cambridge, UK); SOD1, rabbit, polyclonal (Abcam, Cambridge, UK). The secondary antibodies were biotinylated horse anti-rabbit IgG (H+L) (VectorLabs, Orton Southgate, UK), or goat anti-rat IgG2b:horse radish peroxidase conjugate (AbDSerotec, Kidlington, UK) with 3,3′-diaminobenzidine as the substrate (VectorLabs, Orton Southgate, UK). Images are representative of at least two replicates from at least four independent biological replicates. Brown colour indicates positive reaction. Blue colour indicates no staining.

For fluorescent detection of ATP7B, the secondary antibody used was goat anti-rabbit Alexa Fluor 647 (IgG H&L) conjugate (Abcam, Cambridge, UK). The staining was performed in the dark, after which the sections were mounted using VectaShield Mounting Medium with DAPI (VectorLabs, Orton Southgate, UK).

### Gene expression studies

**Fig 1:**
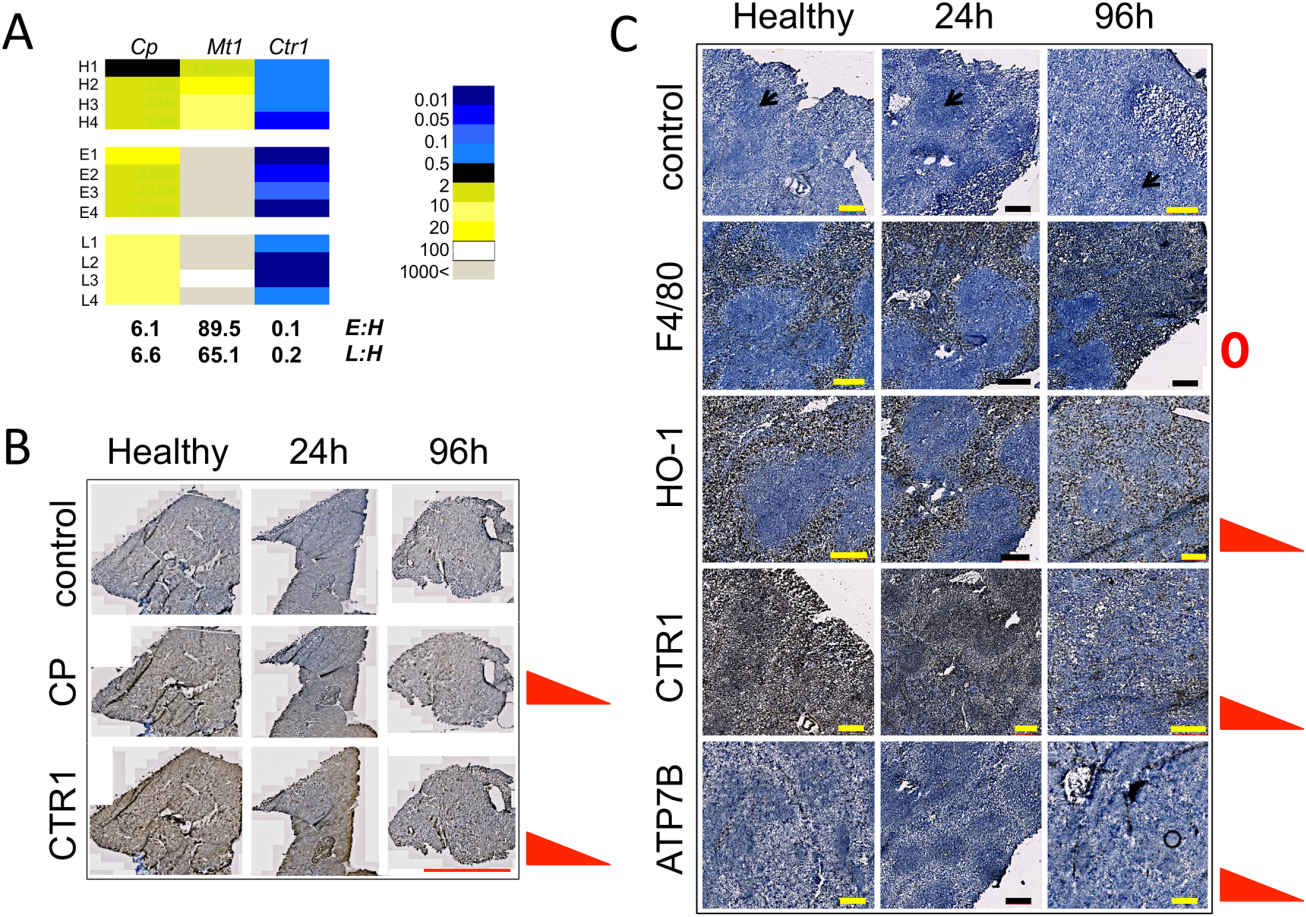
Systemic candidiasis perturbs copper homeostatic functions in the liver and spleen. Progressive *C*. *albicans* infection affects the expression of copper homeostatic functions in the liver at transcript (A) and protein (B) levels. As the infection progresses, the abundance of hepatic transcripts for the metal storage protein metallothionein MT1 and the copper-containing ferroxidase ceruloplasmin (CP) increase, while the abundance of the copper importer *Ctr1* transcript decreases (A). In contrast, hepatic ceruloplasmin protein levels do not increase with progressive *C*. *albicans* infection, while CTR1 protein levels diminish (B), as assessed by immunohistochemistry (Materials and Methods). All transcript data were acquired in duplicate from four biological replicates: healthy mice (replicates H1–H4), animals at early (24 h, replicates E1–E4) or late (96 h, replicates L1–L4) infection stages, following injection with saline (controls, H) or *C*. *albicans* SC5314. Transcript abundances were normalised against the *GAPDH* mRNA. Fold differences in expression are given when *p*≤0.05. Numerical data are provided in S4 Table. (C) In the spleen, the red pulp macrophage population remains relatively constant, as revealed by detection of the red pulp macrophage antigen F4/80. The haem oxygenase HO-1, CTR1 importer and trans-Golgi ATPase ATP7B all decrease in the red pulp over the course of infection. Images are representative of four biological replicates. Brown colour indicates positive reaction and blue colour no reaction. Size bars: 5 mm (B) or 200 μm (C).

**Fig 2:**
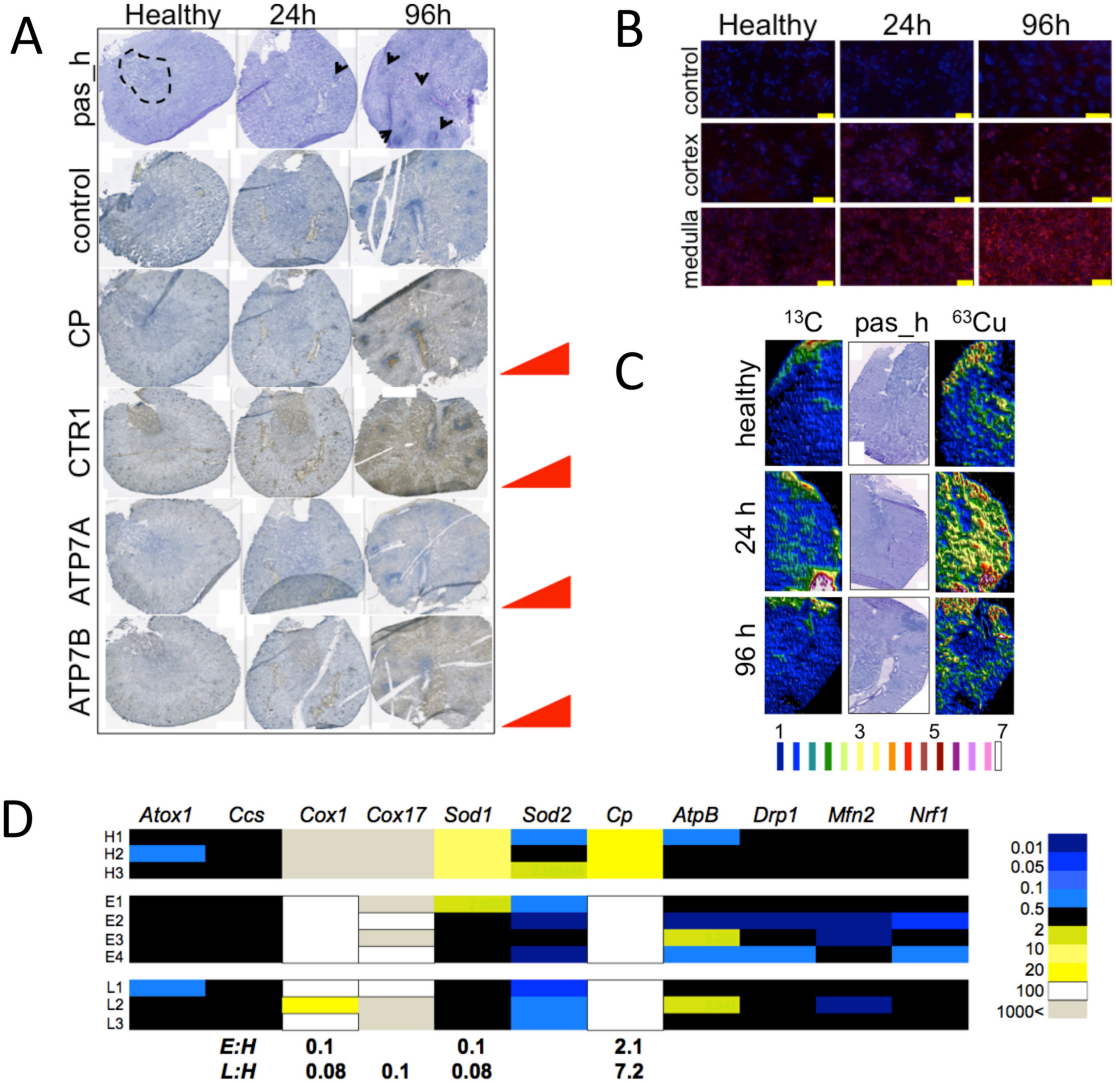
Systemic candidiasis perturbs copper homeostatic functions of the kidney and is accompanied by renal metal redistribution. (A) As the infection develops, renal metabolism shifts towards increased copper acquisition. Immunohistochemistry revealed an increase in CTR1 copper importer, ceruloplasmin (CP), and the trans-Golgi copper transporting ATPases ATP7A and ATP7B, during infection. The location of the renal medulla is indicated inside the dotted area of the top left panel. Arrows mark *C*. *albicans* lesions. Brown colour indicates positive reaction and blue colour no reaction. (B) Fluorescent detection of ATP7B protein with Alexa Fluor 647 antibody conjugate showed a progressive increase in signal (false-coloured red, over the blue DAPI signal) in both the medulla and cortex, with no apparent changes in subcellular distribution of the protein (Size bars: 20 μm). (C) As the *C*. *albicans* infection develops, there is a transient redistribution of copper in renal tissue as shown by ^63^Cu measurements using LA-ICP MS. ^13^C levels remain relatively unchanged throughout, whereas there is a transient increase in tissue ^63^Cu early in the infection (far right). Histology of the corresponding tissue sections is shown (‘pas_h’, middle right). The sequential transverse kidney sections shown in (A-C) are representative of at least two technical replicates, with at least three biological replicates. (D) The shifts in copper distribution are reinforced by changes in the expression of genes encoding copper-associated functions. These transcript abundance data were acquired in duplicate from at least three biological replicates. Transcript abundances were normalised to the *GAPDH* mRNA. Fold differences in expression are given when *p*≤0.05. Numerical data are given in S5 Table.

**Fig 3:**
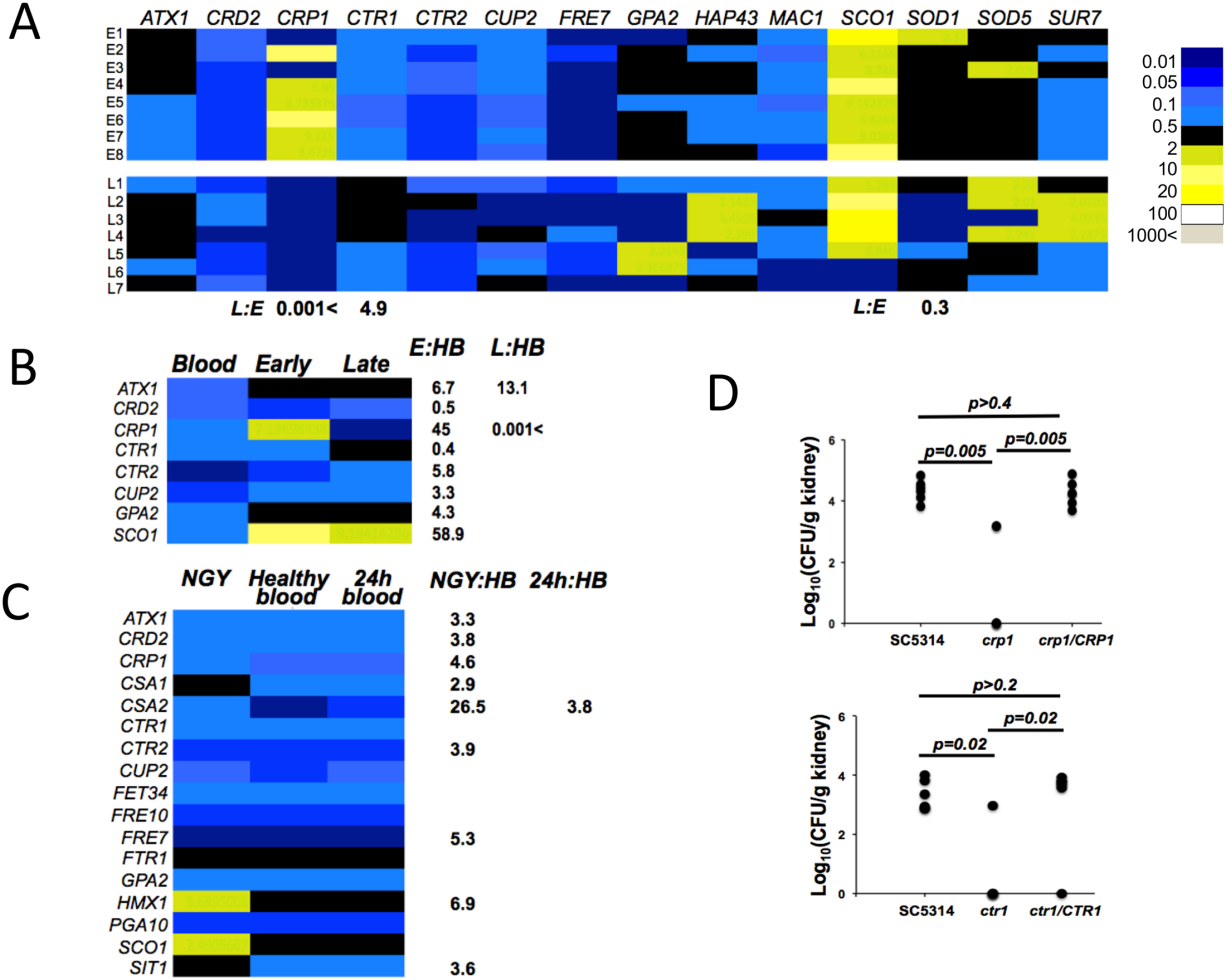
*C*. *albicans* copper homeostasis responds specifically to the changing renal tissue microenvironment during infection. (A) The fungus senses and responds to the copper redistribution in the colonised kidney, as evidenced by the significant up-regulation of the *CRP1* copper exporter gene early in the infection, and the *CTR1* copper importer gene at the later infection stage. These transcript data were acquired in duplicate from at least seven biological replicates: early (24 h, replicates E1–E8); late (96 h, replicates L1–L7) infection stage. Fungal transcript abundances were normalised to the *ACT1* mRNA. Fold differences of expression are given when *p*≤0.05. Numerical data are given in S6 Table. (B) The *C*. *albicans* gene expression pattern in fungal cells isolated from the colonised kidneys is distinct from the fungal response to the blood, supporting tissue-specific response of the *CTR1* and *CRP1* transcripts in (A). Blood was obtained from three healthy animals. Transcript abundances for blood and *in vitro* experiments were acquired in duplicate from three biological replicates. Colour scale is as in (A). Numerical data are given in S7 Table. (C) *C*. *albicans* transcriptional response to the blood from healthy animals (*HB*) and animals 24 h post infection (*24h*) differs from that of *in vitro* (NGY) grown cells, and is largely unchanged for the subset of genes analysed. Colour scale is as in (A). Numerical data are given in S8 Table. (D) The inactivation of either *CRP1* or *CTR1* compromises *C*. *albicans* virulence in the mouse model, as assessed by determining fungal loads in the kidney 72 h post infection. Mann-Whitney U test with two-tailed t-test was used to assess statistical differences between the groups (n = 6 mice per group; the detection limit of our CFU determining method is ca. 100 fugal cells/kidney; CFU counts were below the detection limit in four animals infected with *crp1* null mutant and in five animals infected with *ctr1* null mutant). Differences were considered statistically significant when *p*≤0.05, and the corresponding *p* values are given.

Mouse and fungal transcripts from tissues fixed with RNAlater (Qiagen, Crawley, UK) or *in vitro C*. *albicans* cultures were quantified as described elsewhere [30]. Roche LightCycler 480 and Universal Probes were used in monocolour hydrolyses reactions in qRT-PCR, according to the manufacturer’s instructions. Primer pairs and specific probes are listed in S3 Table and elsewhere [12,30]. Fungal transcript levels were normalised to the *ACT1* mRNA control, and mammalian transcript levels to the *GAPDH* mRNA. Differences were considered statistically significant for *p*≤0.05 in two-tailed t-test, using the Mann-Whitney U test. For multiclass comparisons in Figs 1A, 2D, 3B and 3C and S4 Fig, the Mann-Whitney U test was performed only after Kruskal-Wallis statistics revealed significant differences (*p*≤0.05) between the compared groups.

### Laser ablation inductively coupled plasma mass spectrometry (LA-ICP MS) and ICP-MS for total tissue metal content

LA-ICP MS experiments were performed as described previously [12]. Sequential 22-μm-thick cryosections from the same tissue were prepared for LA-ICP MS, histology and immunohistochemistry. Elemental distribution mapping was performed with a laser ablation system (UP-213, New Wave) coupled to an Agilent 7500c ICP-MS, as described previously [12]. Data were background subtracted and plotted using Microsoft Excel v14.2.4. The images are representative of at least three biological replicates. Total tissue copper measurements were conducted using standard methods [31]. The data are averages from kidneys of four different animals at the indicated infection stages, pooled and measured in samples of two.

### Ethics

All animal experiments were approved by the University of Aberdeen Welfare and Ethical Review Board. The experiments were conducted in compliance with United Kingdom Home Office licenses for research on animals (project license number PPL 60/4135) and in accordance with EU Directive 2010/63/EU. Animals were euthanized by cervical dislocation. All experiments are reported in accordance with the ARRIVE guidelines [32].

## Results

### *C*. *albicans* infections affect hepatic copper metabolism

The mammalian liver is involved in iron and copper homeostasis. It is a major site of iron storage, with ferric iron trapped in ferritin [33], and regulates iron fluxes in other organs through the production and excretion of the soluble hormone hepcidin [34,35]. The liver also produces ceruloplasmin, the major plasma cuproprotein, and ferroxidase that promotes iron mobilisation [13,36]. The liver receives the bulk of dietary copper from the duodenum, conjugated to albumin or α2-macroglobulin. Ceruloplasmin also promotes the delivery of copper to other organs [37,38].

During disseminated *C*. *albicans* infections, hepcidin production and hepatic iron stores increase dramatically [12]. Given the documented relationship between copper and iron homeostases [13], we profiled key hepatic copper-related transcripts to gain insight into the effect of *C*. *albicans* infection upon hepatic copper metabolism. These included transcripts encoding the mammalian copper storage protein metallothionein MT1, the high-affinity copper importer CTR1, and ceruloplasmin. We sampled the livers of healthy animals and those of animals infected systemically for 24 or 96 h (henceforth referred to as ‘early’ or ‘late’ infection stages) (Fig 1A, S4 Table). The *Mt1* transcript was significantly up-regulated while the *Ctr1* transcript was significantly down-regulated at both infection time points. This suggested increased copper storage and decreased copper consumption by the liver [39,40]. Indeed, recently Li *et al* [41] reported perturbations in hepatic copper content during the course of candidiasis infections in mice. Ceruloplasmin production is induced during infections as part of the generic acute phase response of the host [42]. Accordingly, the *Cp* transcript was significantly up-regulated in the livers of infected, but not healthy, animals (Fig 1A). Interestingly, this was not accompanied by an increase in hepatic ceruloplasmin protein levels, as determined by immunohistochemistry (Fig 1B). This might suggest that *Cp* gene expression is regulated at a posttranscriptional level [43]. CTR1 protein levels decreased as infection progressed, which correlated with the decline in *Ctr1* transcript levels (Fig 1A and 1B). We conclude that systemic *C*. *albicans* infection perturbs host copper homeostasis in the liver.

### *C*. *albicans* infections perturb splenic copper homeostasis

We showed previously that systemic candidiasis affects metal (iron) metabolism in the spleen [12]. Therefore, we investigated the effects of *C*. *albicans* infection on copper appropriating proteins of the splenic red pulp. We used specific antibodies to probe splenic levels of ceruloplasmin, CTR1 and ATP7B (a trans-Golgi copper efflux ATPase which supplies copper to various cuproproteins [13,44]). While ceruloplasmin was barely detectable and remained unchanged (not shown), both CTR1 and ATP7B decreased over the course of systemic infection (Fig 1C). These data are consistent with a lowered splenic copper consumption. Indeed, splenic copper levels were recently shown to decrease during candidiasis in the mouse model [41], further supporting the view that copper appropriation by the spleen declines during disseminated *C*. *albicans* infections.

In certain cell types, depending on copper availability, ATP7B and the closely related copper transporter ATP7A relocalise to facilitate copper export rather than trans-Golgi delivery, migrating to the apical or basolateral membranes, respectively [13,45,46]. We observed no change in the subcellular localisation of ATP7B, which was detected as defined punctate structures at each infection stage (S1 Fig). However, we did observe a gradual decrease in ATP7B levels in the splenic red pulp, suggesting quantitative rather than qualitative effects of infection on ATP7B.

Collectively, our data indicate readjustments of hepatic and splenic copper homeostatic functions during the development of systemic *C*. *albicans* infections.

### Adjustment of renal copper homeostasis coincides with increased renal iron acquisition during *C*. *albicans* infections

During disseminated candidiasis, the kidney takes over some erythrocyte recycling functions from the spleen, and during the latter stages of infection this is reflected in increased renal transferrin receptor (iron acquisition), HO-1 and HO-2 (haem iron extraction), and hepcidin and ferritin (iron retention) [12]. Renal iron medullary deposits increase as a result. Simultaneously, the host imposes iron nutritional immunity in the renal cortex through infiltrating immune cells [12]. Given the well-documented mechanistic link between the iron and copper homeostases [13], we reasoned that kidney copper metabolism would also be affected by systemic candidiasis. Therefore, we compared renal ceruloplasmin, CTR1, ATP7A and ATP7B protein levels in animals at different stages of infection (Fig 2A). Indeed, the levels of these copper homeostasis proteins increased during infection. Furthermore, the abundance of ATP7B progressively increased in both the renal cortex and medulla during infection, but its subcellular localisation remained unchanged over the course of the disease (Fig 2B). Although ATP7B is generally thought to be liver-specific, this result is consistent with a previous report on ATP7B dynamics in an *in vitro* model [46]. Collectively, these data suggest increased renal copper acquisition and/or redistribution during systemic fungal infection.

We next mapped ^63^Cu in kidneys from animals at different stages of infection and observed a transient increase in the overall copper in early infection (Fig 2C). We measured the total amount of kidney copper from organs of healthy animals, and animals at early and late infection stages. There was a ca. 10% increase in total kidney copper at the 24 h time point, when compared with healthy animals (16.6±0.5 mg/kg dry weight ‘early infection’ vs. 15.3±0.8 mg/kg dry weight ‘healthy’). Late in the infection, the total kidney copper levels fell by more than ca. 10% compared to the healthy controls (13.9±0.4 mg/kg dry weight ‘late infection’). The recent data of Li *et al* [41] reinforce these observations. The kidney is more resilient to global metal content changes than other organs [47], and therefore the detected changes in copper may suggest a physiologically relevant role for the infection process.

Our data indicated changes in renal copper loading and distribution during disease progression, suggesting possible alterations in intracellular renal copper pools. We therefore profiled the expression of mouse genes encoding copper metallochaperones and some of their clients: *Ccs* and *Sod1*; *Cox17* and *Cox1*; *Atox1* for *Atp7B*; ceruloplasmin (*Cp*), the main cuproprotein involved in iron loading of transferrin; and the mitochondrial Mn *Sod2* (Fig 2D, S5 Table). The abundances of the *Cox1*, *Cox17* and *Sod1* transcripts all decreased in abundance during disease progression. Interestingly, SOD1 protein was present at lesion sites late in the infection (S2 Fig). *Ccs* and *Sod2* transcript levels remained unchanged. Remarkably, *Cp* transcript was up-regulated about seven-fold in infected kidney, relative to the healthy controls. This suggests that the ceruloplasmin protein detected late in the infection (Fig 2A) may have been synthesised by the kidney [48], rather than being transported from the liver. This corroborated our observation that hepatic ceruloplasmin levels do not increase during the course of infection (Fig 1B).

Since the kidney is relatively resilient to changes in metal content compared to other organs [47], we surmised that changes in *Cox1* and *Cox17* mRNA levels during infection could either signify kidney injury or infection-triggered shifts in renal copper dynamics. Mitochondrial dysfunction and fragmentation occur during kidney injury [49,50]. However, *Cox1* transcript levels decreased early, i.e., 24 h post infection, when only modest numbers of *C*. *albicans* cells were detectable in the kidneys. Furthermore, the mRNA levels for genes encoding mitochondrial fission and fusion proteins remained unchanged during the infection (Fig 2D). Thus, the changes in renal copper homeostasis gene expression during systemic *C*. *albicans* infection appear to reflect an active response to infection rather than tissue damage.

### Renal copper fluxes trigger micronutrient adaptation in *C*. *albicans* during systemic infection

The detected changes in renal copper content during disseminated *C*. *albicans* infections (Fig 2C) temporally coincided with the mobilisation of renal iron toward the medulla and away from fungal lesions during the infection [12]. We previously showed that the fungus senses the reduction in iron availability and responds by activating haem iron acquisition alongside its reductive iron acquisition pathway [12]. Therefore, we reasoned that the dynamic cupric/cuprous renal microenvironment should also be detected by the fungus. We examined the expression of copper-associated genes in fungal cells from renal lesions, focusing on copper transporters, chaperones, regulators and storage proteins (Fig 3A, S6 Table). Transcript levels for the high affinity copper efflux ATPase Crp1 [51] were significantly higher at early infection stages, and then declined. This was consistent with a recent report indicating that fungal *CRP1* expression increases early during systemic infection [52]. In contrast, expression of the high affinity copper importer *CTR1* [53] was lower at early infection stage and then increased during the course of infection (Fig 3). These expression patterns differed from those of *C*. *albicans* cells exposed to blood (Fig 3B, S7 Table) or those grown *in vitro* (Fig 3C, S8 Table). Rather, they were consistent with fungal copper efflux coinciding with the transient increase in renal copper levels early in infection. Furthermore, the data suggested an increase in fungal copper import in response to the subsequent decline in renal copper levels (Fig 2C).

We examined the regulation of *C*. *albicans CRP1* (copper efflux pump) and *CTR1* (copper importer) in response to copper *in vitro*. *CRP1* gene expression increased while *CTR1* expression decreased in response to elevated copper concentrations, in agreement with earlier reports [51,53,54] (S3 Fig). In addition, *CTR1* was induced under iron limitation, consistent with the interaction of iron and copper micronutrients and in agreement with previous studies [55] (S4 Fig, S9 Table). Regulation of these *C*. *albicans* genes in response to copper and iron *in vitro* reflected their expression patterns *in vivo* in response to the observed changes in renal metal ion availability (Fig 2C and Potrykus *et al* [12]).

We next asked whether the *C*. *albicans* cells were responding to copper content in the bloodstream or in the renal tissue. The bloodstream contains copper-containing entities such as ceruloplasmin, macroglobulin, albumin and amino acids [13], and therefore it was possible that the *C*. *albicans* cells were reacting to copper levels in the blood. Indeed, a progressive increase in serum copper has been reported over the course of systemic candidiasis in the mouse model [41]. Hence we examined the impact of blood from infected mice and healthy controls upon the expression of copper- (and also iron-) related genes in *C*. *albicans*. *C*. *albicans* cells were incubated for 30 min with blood from healthy animals and animals 24 h post-infection (Fig 3C) as this time point coincided with high *CRP1* expression levels *in vivo* (Fig 3A) and fungal cells *in vitro* respond to copper within this timescale (S4 Fig). Significant differences were observed between the blood-incubated samples and the corresponding *in vitro* controls for most of the genes analysed. However, the only gene displaying a significant difference between *C*. *albicans* cells exposed to infected or uninfected blood was *CSA2*, which encodes a haem receptor (Fig 3C). Given the minimal differences between infected and uninfected blood, we inferred that the observed changes in renal copper distribution during systemic candidiasis, and the accompanying *C*. *albicans* gene expression patterns, reflect changes in copper retention by the kidney rather than bloodstream copper accessible to the fungus.

### The virulence of *C*. *albicans* during systemic infection depends on both *CRP1* and *CTR1*

The *CRP1* and *CTR1* genes encoding the copper efflux pump and importer, respectively, are differentially regulated during systemic infection (Fig 3A). To test whether these genes are important for infection, we constructed isogenic *C*. *albicans* deletion mutants in the genetic background of the clinical isolate SC5314 [33] using our *Clox* gene disruption system [36] and tested their virulence in the mouse model of systemic infection [12]. We inoculated mice with similar numbers of fungal cells, based on haemocytometer measurements of total cell numbers. Subsequent measurements of the actual CFUs revealed differences in the number of live fungal cells injected into the mice possibly due to different strain viabilities *in vitro*. This might have affected the final kidney CFU outcomes. Nevertheless, the data strongly suggest that *CRP1* and *CTR1* were both required for the full virulence of *C*. *albicans* (Fig 3D). Thus, during infection, *C*. *albicans* cells require the capacity to extrude, as well as import and assimilate, copper.

**Fig 4:**
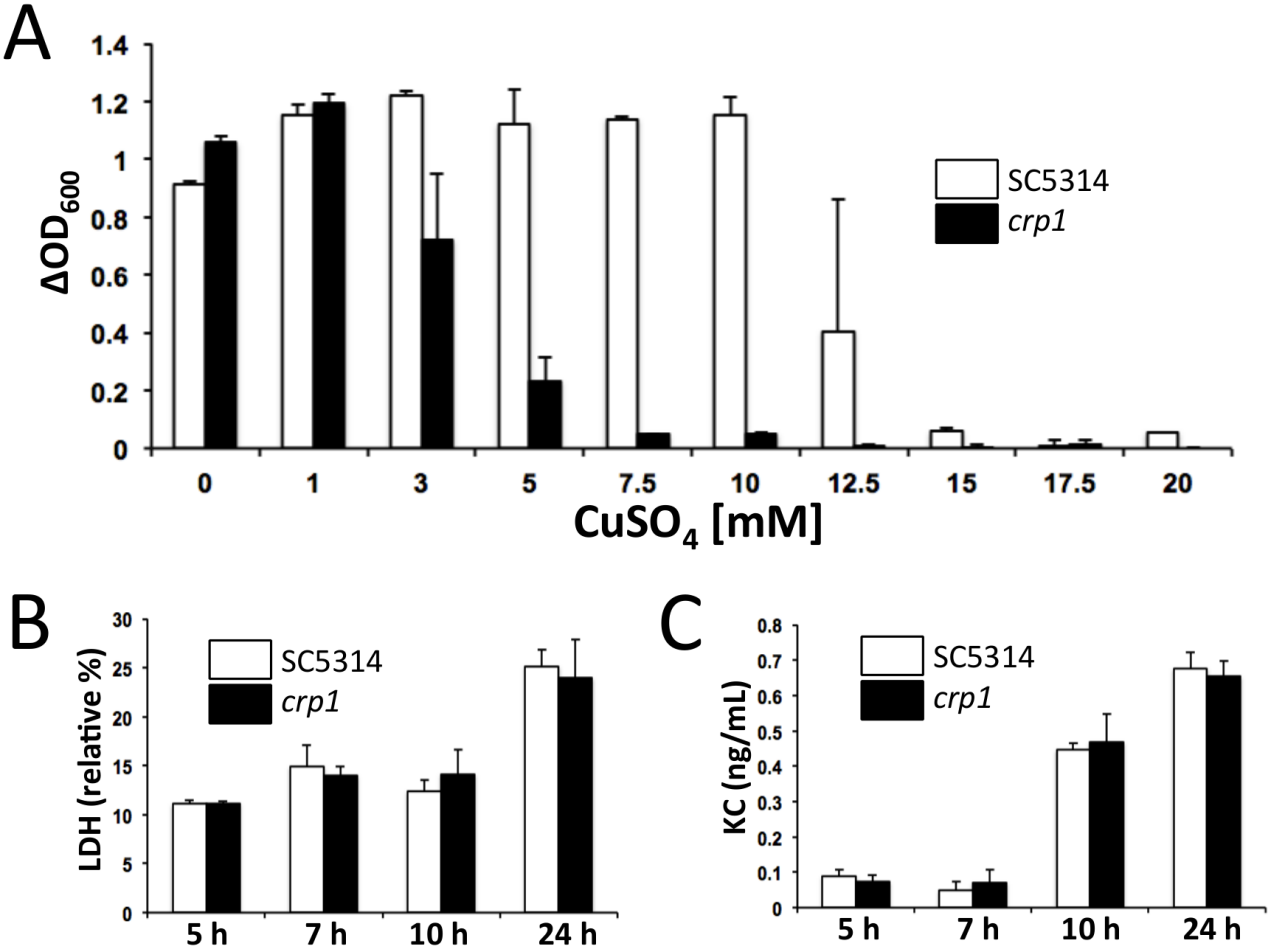
The *C*. *albicans CRP1* copper exporting ATPase is required for copper tolerance *in vitro* and plays a role in fungus-host interaction *in vivo*. (A) The deletion of *CRP1* severely impairs the ability of *C*. *albicans* to grow in the presence of copper. *C*. *albicans* cells were grown at 30°C overnight in YPD medium. Cultures were diluted at OD600 = 0.001 into fresh medium containing the appropriate CuSO_4_ concentrations, and growth was monitored after 21 h, at 30°C. The values represent differences between the final and initial OD600 of cultures (+/- SD from two technical replicates), and are representative of three separate experiments performed. (B) Nevertheless, *CRP1* deletion does not impair the interaction of *C*. *albicans* with murine renal epithelial cells *in vitro*. The *CRP1* mutant elicits a similar degree of damage to the renal epithelial cell monolayer as the parental strain SC5314, as assessed by measurements of LDH release. At least three independent *C*. *albicans* inocula were used in at least two independent co-incubation experiments, in duplicate. (C) Similarly, there is no difference in the release of KC by the renal cells when incubated with the *crp1* mutant versus the SC5314 isogenic control.

The main phenotype of the *CRP1* null mutant *in vitro* was its exquisite sensitivity to copper. In rich YPD medium, *crp1* cells were unable to grow at Cu^2+^ concentrations over 7.5 mM, whereas wild type cells tolerated concentrations of 12.5 mM (Fig 4A). This recapitulated the phenotype of *C*. *albicans crp1* mutants constructed in other genetic backgrounds [51,54].

We tested whether the virulence defect of *crp1* cells resulted from its role in the *C*. *albicans*-renal cell interaction. To achieve this we exploited our *ex vivo* model of renal infection, which is a proxy for *C*. *albicans* virulence studies [29]. The interaction of *C*. *albicans* with the renal epithelial cells in this *ex vivo* model was not compromised by *CRP1* inactivation (Fig 4B and 4C). No significant differences between *C*. *albicans crp1* and wild type cells were observed with respect to their ability to stimulate lactate dehydrogenase (LDH) release or KC cytokine production by the renal cells (Fig 4B and 4C). Furthermore, after an overnight incubation, the *crp1* mutant formed a matt of fungal cells over the renal cells, comparable to the wild type control (not shown). Therefore, the transient *CRP1* up-regulation observed *in vivo* during developing systemic infection (Fig 3A) is not simply an outcome of fungus-renal cell interactions.

**Fig 5:**
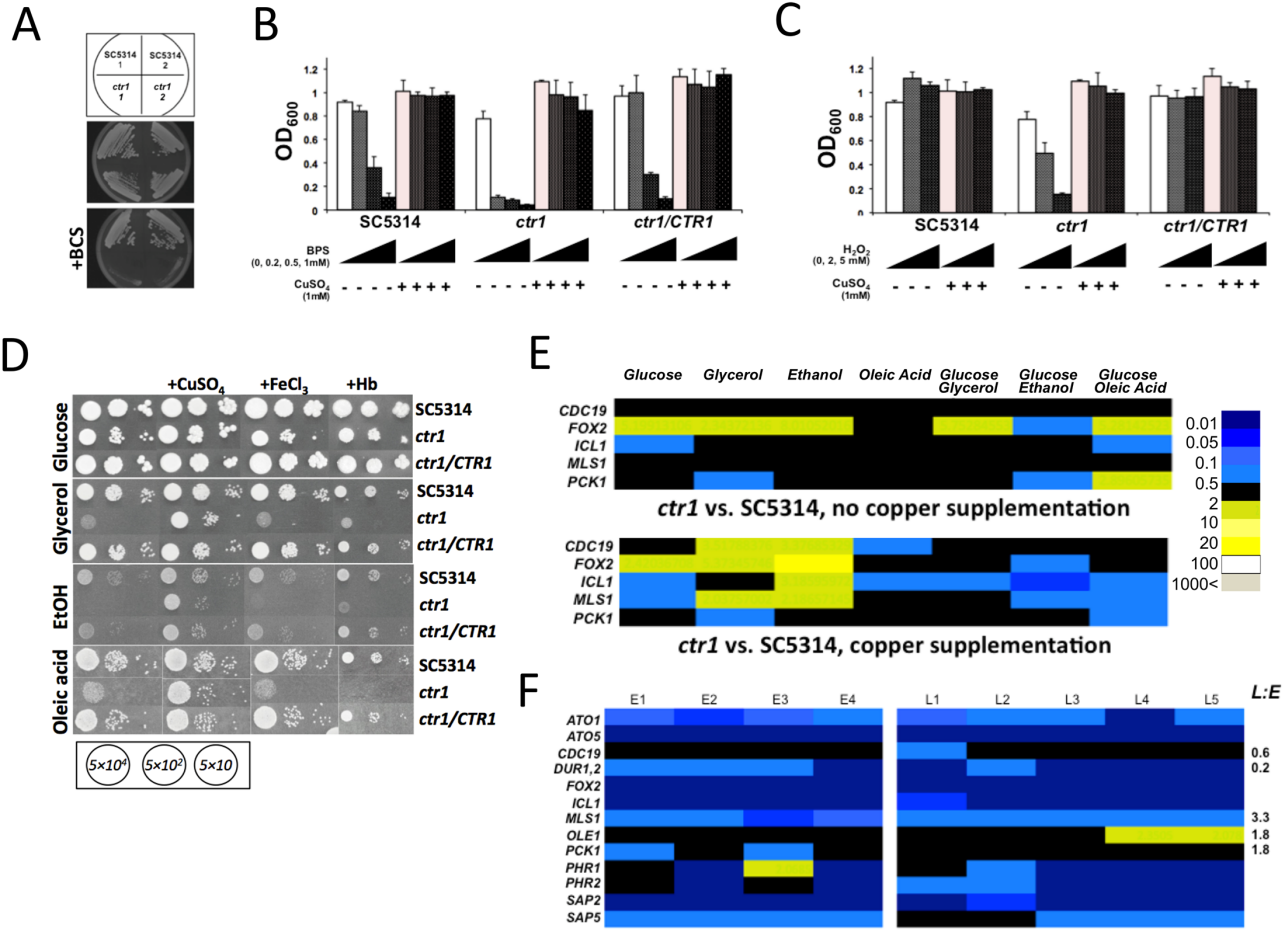
High affinity copper acquisition influences fungal pathogenicity factors. *C*. *albicans ctr1* cells are susceptible to copper deprivation imposed using the copper chelator BCS (95 μM) (A). The *C*. *albicans ctr1* mutant is also sensitive to iron chelation by BPS (B). The *C*. *albicans ctr1* strain is highly sensitive to H_2_O_2_ (C). Both of these defects are reversed by copper supplementation. Further, the *C*. *albicans ctr1* mutant displays growth defects on various carbon sources, a phenotype that is suppressed by copper supplementation, but not by haem or Fe^3+^ addition (D). Images were taken after 72 h incubation at 30°C. Number of cells spotted in every subset is given at the bottom of the panel. (E) The transcript levels for key metabolic genes are perturbed in *ctr1* cells, in the absence (top) and presence (bottom) of copper. These data represent averages of three biological replicates with two technical replicates. Numerical data are given in S10 Table. (F) Changes in *C*. *albicans* transcript levels during renal colonisation. These data represent duplicate measurements from at least four biological replicates from the kidneys of animals at early (24 h, replicates E1–E4, 4 animals) or late (96 h, replicates L1–L5, 5 animals) infection stage. Colour scale is as in (E). Fungal transcript abundances were normalised to the *ACT1* mRNA. Numerical data are given in S11 Table.

The deletion of the Ctr1 importer impaired copper acquisition by the fungus and also exerted pleiotropic effects on *C*. *albicans* (Fig 5). Deletion of *CTR1* in the SC5314 background rendered *C*. *albicans* cells sensitive to copper deprivation (Fig 5A), thereby recapitulating the phenotype of *ctr1* cells in a different, *ura3-*, genetic background [53]. Furthermore, the *ctr1* null mutant was sensitive to iron chelating agents (Fig 5B), thereby underscoring the role of copper in iron acquisition [55]. We note that while BPS is generally viewed as an iron-specific chelator, it can also bind other metals. Therefore, we do not exclude the possibility that the chelation of other metals may contribute to the observed effects of BPS. Mutant *ctr1* cells were also sensitive to oxidative (H_2_O_2_) stress. Interestingly, these phenotypes were suppressed by copper supplementation, indicating that they were caused by intracellular copper deprivation. Iron scavenging and oxidative stress resistance promote *C*. *albicans* pathogenicity [56–58], and, therefore, it was not surprising that *ctr1* cells displayed attenuated virulence during systemic infection (Fig 3D).

Interestingly, *C*. *albicans ctr1* cells failed to utilise glycerol or ethanol, and their ability to assimilate glucose and oleic acid was also compromised (Fig 5D). These defects in carbon assimilation were rescued by copper supplementation, but not by ferric ions or haemoglobin iron (Fig 5D). Thus they were mediated by copper, not iron, insufficiency resulting from the lack of the high affinity copper importer. Furthermore, the growth defects of *C*. *albicans ctr1* cells were accompanied by changes in core metabolic pathway gene expression (Fig 5E, S10 Table). It has been established that *C*. *albicans* dynamically readjusts its core metabolism during the course of systemic infections. For example, the *CDC19* gene encoding the glycolytic enzyme pyruvate kinase is down-regulated, while transcripts for the gluconeogenic enzyme phosphoenolpyruvate carboxykinase (*PCK1*) and the glyoxylate cycle enzyme malate synthase *(MLS1*) are up-regulated (Fig 5F, S11 Table) [52,59,60]. Indeed, it has been demonstrated that the ability to assimilate alternative carbon sources is critical for fungal virulence [59,61,62]. Therefore, the influence of Ctr1-mediated copper sequestration upon *C*. *albicans* virulence is probably mediated in part through promotion of efficient carbon assimilation.

## Discussion

Infection sites comprise perpetually changing microcosms, shaped by the dynamic interactions between the pathogen and the host. Micronutrients play a central role in this dynamic interplay during disease progression. Consequently, nutritional immunity has evolved as a mechanism by which the host manipulates local micronutrient concentrations to the detriment of the invading microbe in an attempt to contain an infection [4,9]. The case for nutritional immunity is well documented for iron, and bacterial and fungal pathogens including *C*. *albicans* require effective iron acquisition mechanisms for full virulence [56,58]. Here we demonstrate another layer of complexity governing the pathogen-host tug of war—the regulation of copper homeostasis.

Our data clearly indicate that disseminated fungal infections affect copper metabolism in organs that promptly clear the infection (the liver and spleen), as well as organs that become colonized (the kidney). These changes coincide with previously described [12] infection-associated perturbations in iron metabolism in these organs. Unsurprisingly, the local changes in copper levels are sensed by *C*. *albicans*. Similarly to iron acquisition [12,56,58], intact copper acquisition pathways (Fig 3D) are required for full *C*. *albicans* virulence. Early in the infection, high expression of fungal Crp1 high affinity efflux pump accompanies increased copper levels, possibly to evade copper poisoning [51] (Fig 3). As immune infiltrates create zones of iron-exclusion around fungal cells and iron becomes limiting for the fungus [12], *C*. *albicans* increases the expression of its high affinity copper importer Ctr1. It is tempting to speculate that the down-regulation of fungal copper-dependent reductive iron acquisition in the course of infection [12] is partly driven by changes not just in iron but also copper availability. Access to copper also impacts other key factors that enhance the virulence of *C*. *albicans*, namely, oxidative stress resistance [57,63,64], carbon assimilation and the expression of core metabolic genes [42,59,65–67].

In conclusion, we demonstrate the impact of systemic infection on copper homeostasis in the host, the interplay between copper and other essential micronutrients such as iron in infected tissues *in situ*, and the effects of host copper on fungal copper homeostasis and carbon assimilation. It is clear that “nutritional immunity” is more than just a static limitation of a single nutrient. Rather, this phenomenon is a cumulative outcome of dynamic, simultaneous spatio-temporal alterations of multiple micronutrients impacting the progression of infection.

## Supporting Information

S1 Fig Subcellular localisation of splenic copper trans-Golgi transporting ATPase ATP7B is unchanged during *C*. *albicans* infection. As the infection progresses, ATP7B protein decreases in the red pulp of the spleen, as determined by immunoflourescent probing with Alexa Fluor 674 labelled antibodies. The subcellular localisation of the protein does not change (arrows). Tissue sections in the Figure are sequential to those in Fig 1C. The staining is representative of results from four biological replicates. Red, ATP7B (arrows). Blue, DAPI counterstain. Size bars, 20 μm.
(TIF)

S2 Fig During development of systemic candidiasis, renal superoxide dismutase SOD1 localises to fungal lesion sites (black arrows). The SOD1-positive regions correspond to areas occupied by the immune infiltrates, suggesting staining of mouse and not fungal SOD1. The staining is representative of transverse kidney sections from three biological replicates per infection stage. Pas_h, Periodic acid/Schiff staining.
(TIF)

S3 Fig *C*. *albicans CRP1* copper efflux gene and *CTR1* copper importer gene are conversely regulated with changing copper concentrations. Fungal cells were incubated at 30°C for 1h in YPD supplemented with increasing concentrations of CuSO_4_. The relative transcript abundances are normalised to *ACT1*. The values are averages of duplicate measurements performed in duplicate. See Materials and Methods for details. Primary y-axis, normalised *CRP1* abundance; secondary y-axis, normalised *CTR1* abundance.
(TIF)

S4 Fig The effect of copper and iron chelation on the expression of *C*. *albicans* genes encoding core metabolism and metal homeostatic functions. Early exponential phase *C*. *albicans* SC5314 cells grown at 30°C in YNB-Glucose medium were exposed to 170 μM iron chelator BPS or 250 μM copper chelator BCS for 30 min, before fixing in RNAlater. Gene expression was analysed by qRT-PCR, as described in Materials and Methods. Relative transcript abundances are normalised to *ACT1*, and expressed as ratios to control (i.e., no iron or copper chelation). Values are averages of duplicate measurements from three independent experiments. Numerical data are given in S9 Table.
(TIF)

S1 Table Oligonucleotide primers used in construction of *C*. *albicans* mutant strains. (DOCX)

S2 Table Oligonucleotide primers and UPL probes for ploidy verification of *C*. *albicans* mutant strains. (DOCX)

S3 Table Oligonucleotide primers and UPL probes for fungal and mammalian gene expression analyses. (DOCX)

S4 Table Normalised qRT-PCR data for Fig 1A. (XLSX)

S5 Table Normalised qRT-PCR data for Fig 2D. (XLSX)

S6 Table Normalised qRT-PCR data for Fig 3A. (XLSX)

S7 Table Normalised qRT-PCR data for Fig 3B. (XLSX)

S8 Table Normalised qRT-PCR data for Fig 3C. (XLSX)

S9 Table Normalised qRT-PCR data for S4 Fig. (XLSX)

S10 Table Normalised qRT-PCR data for Fig 5E. (XLSX)

S11 Table Normalised qRT-PCR data for Fig 5F. (XLSX)

## Abbreviations

BCS: bathocuproinedisulfonic acid
BPS: bathophenanthrolinedisulfonic acid
C. albicans: *Candida albicans*
Cp: ceruloplasmin transcript
DAPI: 4',6-diamidino-2-phenylindole
ICP MS: inductively coupled plasma mass spectrometry
LA ICP MS: laser ablation ICP MS
qRT-PCR: quantitative real time PCR

Unless otherwise indicated, all chemical reagents were from Sigma Aldrich (Gillingham, Dorset, UK).
