# Supplementary figures and images for: Host-Imposed Copper Poisoning Impacts Fungal Micronutrient Acquisition during Systemic Candida albicans Infections
Source: PLoS One. 2016 Jun 30;11(6):e0158683. doi: 10.1371/journal.pone.0158683 (PMC4928837; doi:10.1371/journal.pone.0158683)

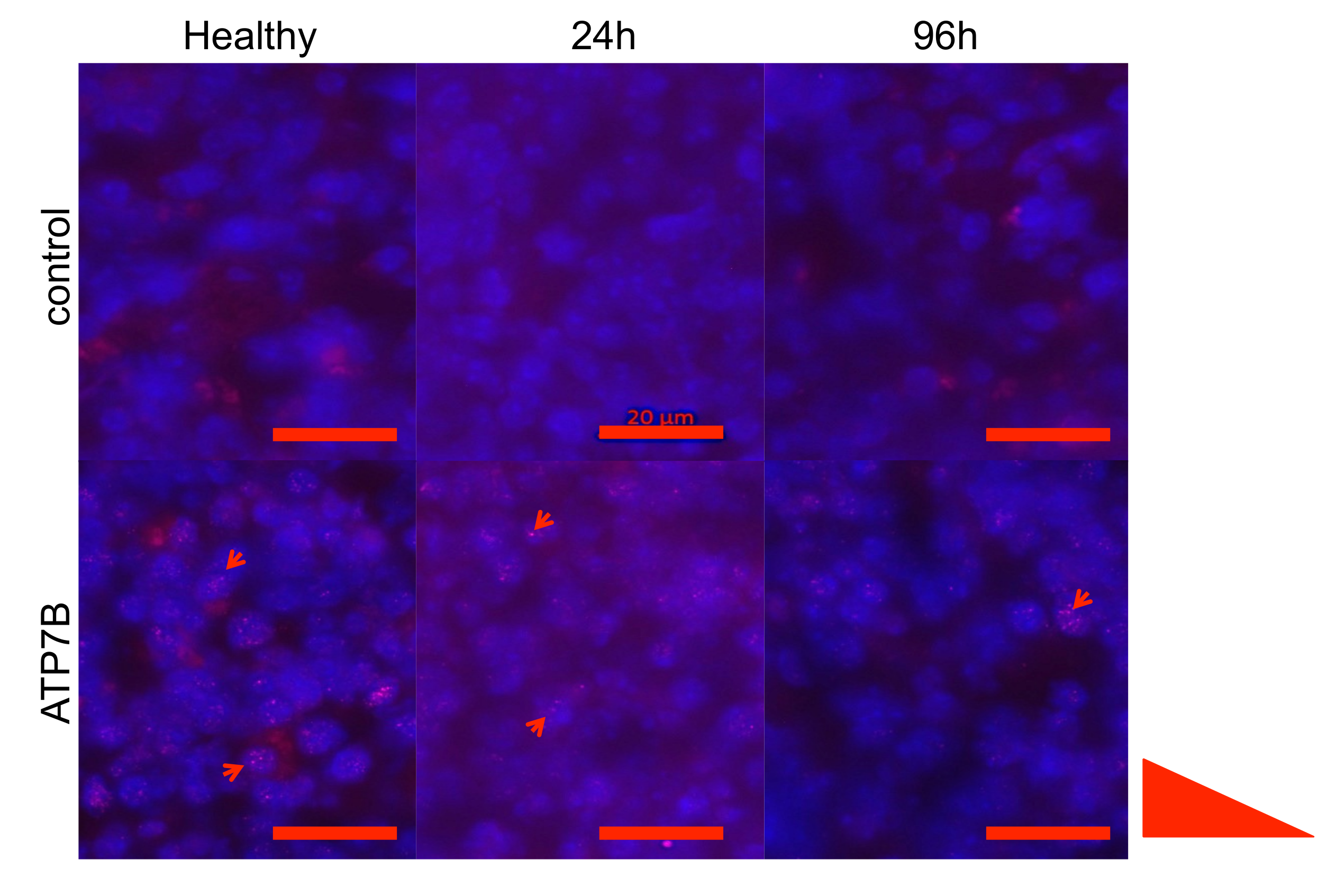

Supplement: S1 Fig — As the infection progresses, ATP7B protein decreases in the red pulp of the spleen, as determined by immunoflourescent probing with Alexa Fluor 674 labelled antibodies. The subcellular localisation of the protein does not change (arrows). Tissue sections in the Figure are sequential to those in Fig 1C. The staining is representative of results from four biological replicates. Red, ATP7B (arrows). Blue, DAPI counterstain. Size bars, 20 μm. (TIF) [file pone.0158683.s001.tif]

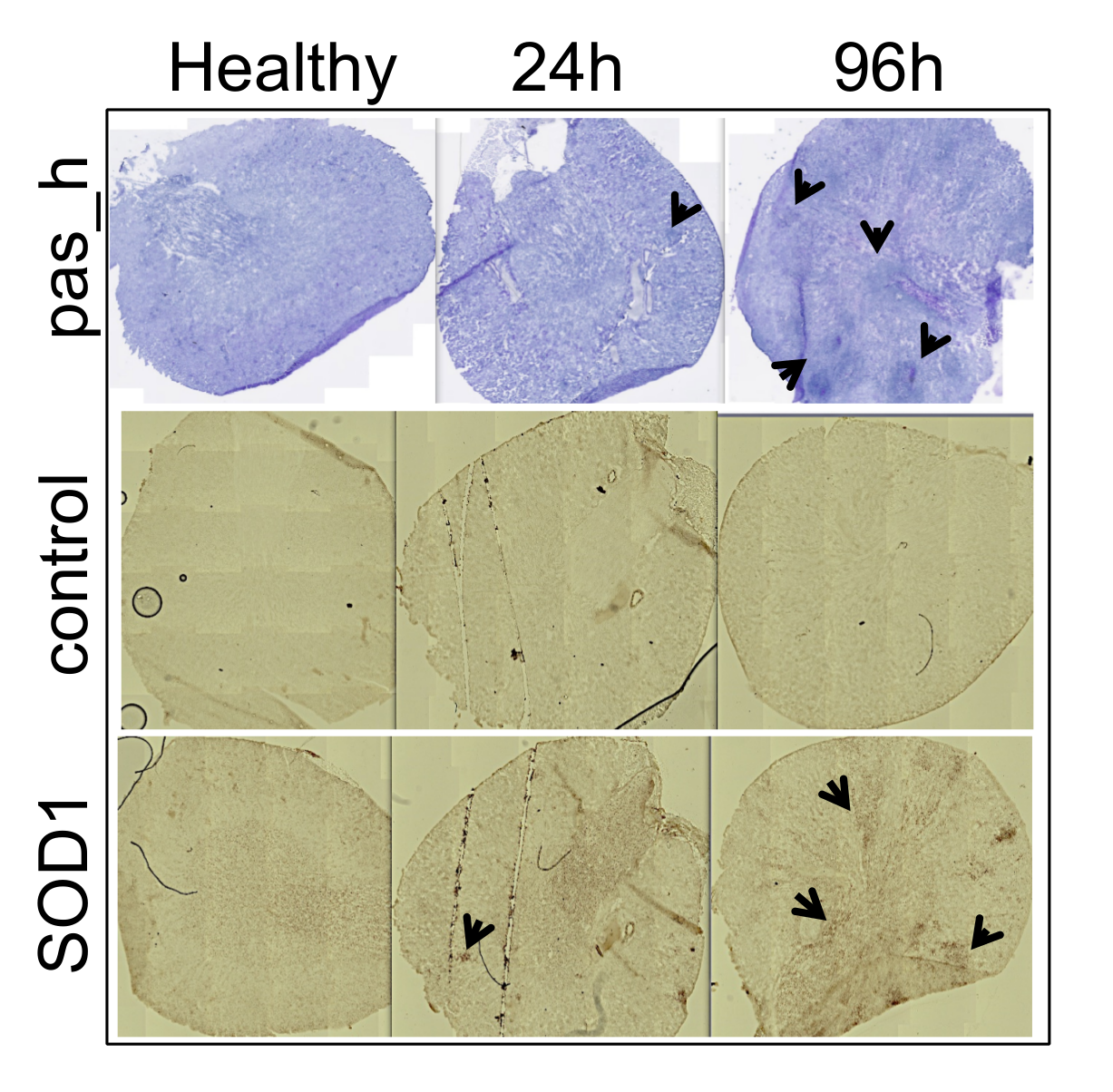

Supplement: S2 Fig — The SOD1-positive regions correspond to areas occupied by the immune infiltrates, suggesting staining of mouse and not fungal SOD1. The staining is representative of transverse kidney sections from three biological replicates per infection stage. Pas_h, Periodic acid/Schiff staining. (TIF) [file pone.0158683.s002.tif]

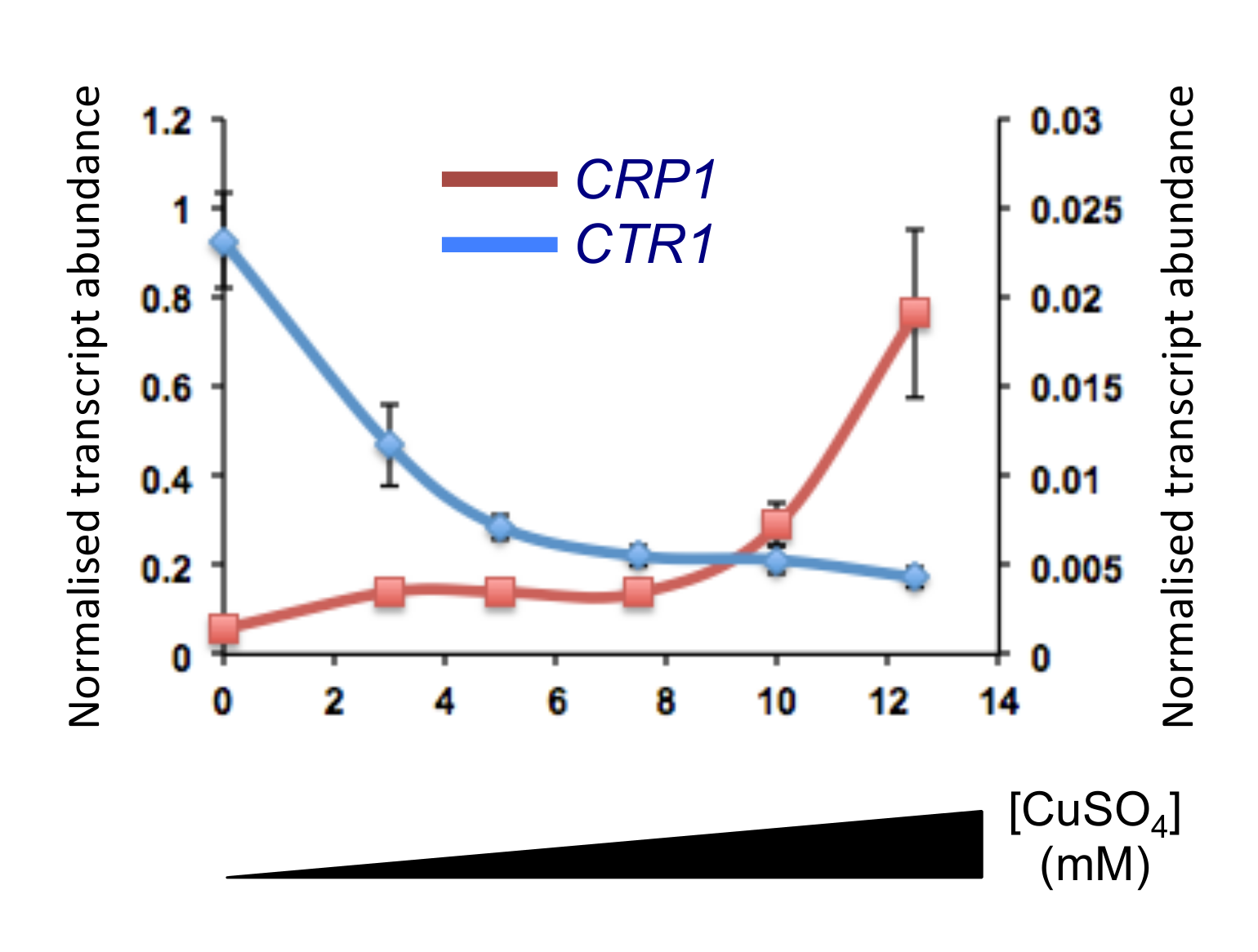

Supplement: S3 Fig — Fungal cells were incubated at 30°C for 1h in YPD supplemented with increasing concentrations of CuSO4. The relative transcript abundances are normalised to ACT1. The values are averages of duplicate measurements performed in duplicate. See Materials and Methods for details. Primary y-axis, normalised CRP1 abundance; secondary y-axis, normalised CTR1 abundance. (TIF) [file pone.0158683.s003.tif]

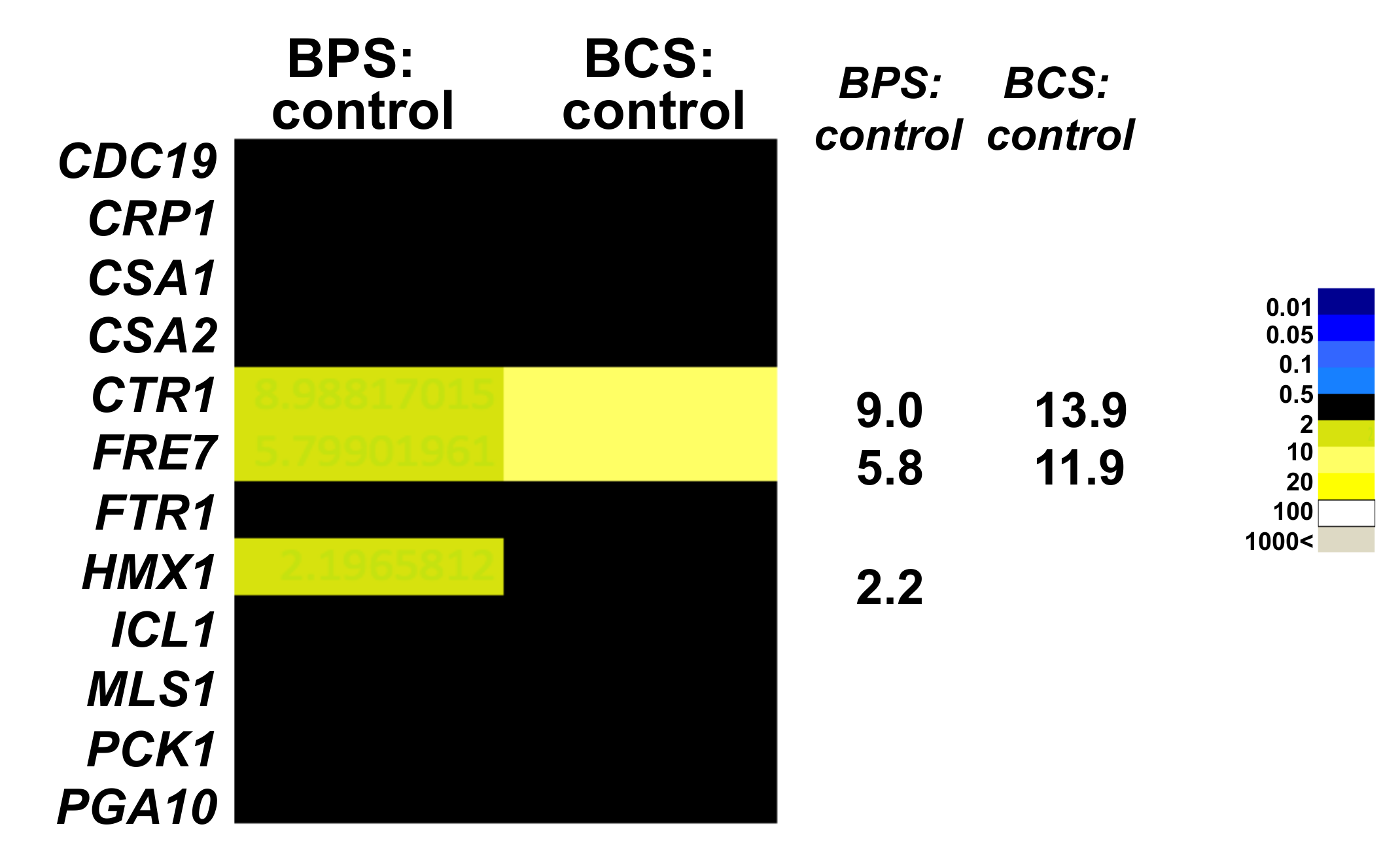

Supplement: S4 Fig — Early exponential phase C. albicans SC5314 cells grown at 30°C in YNB-Glucose medium were exposed to 170 μM iron chelator BPS or 250 μM copper chelator BCS for 30 min, before fixing in RNAlater. Gene expression was analysed by qRT-PCR, as described in Materials and Methods. Relative transcript abundances are normalised to ACT1, and expressed as ratios to control (i.e., no iron or copper chelation). Values are averages of duplicate measurements from three independent experiments. Numerical data are given in S9 Table. (TIF) [file pone.0158683.s004.tif]
