# Supplementary material for: Host-Imposed Copper Poisoning Impacts Fungal Micronutrient Acquisition during Systemic Candida albicans Infections
Source: PLoS One. 2016 Jun 30;11(6):e0158683. doi: 10.1371/journal.pone.0158683 (PMC4928837; doi:10.1371/journal.pone.0158683)
Supplement: S1 Table — (DOCX) [file pone.0158683.s005.docx]

**S1 Table. Oligonucleotide primers used in construction of *C. albicans* mutant strains.**

| Primer Name | Sequence^a^ |
| --- | --- |
| CTR1_CLOx_F_JP | TTGGAAATTCCTTATTTTAATTTAAGTTACATATTATCAACACCACTAATTAGATATATTTTCTTCATTTATCAGTCAACACCACATTTAACCTTACATTACGGCCAGTGAATTGTAATA |
| CTR1_CLOx_R_JP | TTAAAAAATTATTATGTAAACTGTATTTTAAAGAATTATAATTATATGATTATACTCTAAAAAAAAAACAAAGTAATTTTCTTTTTTATGTTTAGTTGTTTCGGAATTAACCCTCACTAA |
| CTR1_CLOx_F3_JP | ATCGGGTATGGCTATGGAAGGTATGGACCACGGTTCTTCTCACATGGCAATGAACATGTGGCTTACAGCTTCATTTAAGGATTATCCTGTTGTGTTCAAAGATTTAAGAGCTACGGCCAGTGAATTGTAATA |
| CTR1_CLOx_R3_JP | AAGGAGTAAGCAAACAAATCTGGAATAATACATAATGCAAGTCTAATAATATCTCTAGAAATAGTTGATGCCAAAGATAATGATTTACCGGTTCCACGAGCTTTTGTGTCGGAATTAACCCTCACTAA |
| SpeI_F_CTR1_ReINT_JP | ATCGACCTTATGACTAGTAAAAGCATTTATTACATCTGCATTTCGTATTTGATGACC |
| Not1_R_CTR1_ReINT_JP | AGAGACAAAACCTTGGCGGCCGCGAGATATGAATTGAGGTGTAAAGCAGTATATTTTGTC |
| CRP1_CLOx_F_JP | AACTTAAAAGTATATAAGACGATCGATATGCCCATTATATAGCATTTCCTTTTCATATTAAATAACTTTCAACCTTTTATCAATAACCTAAGTCCACATAACGGCCAGTGAATTGTAATA |
| CRP1_CLOx_R_JP | TGTTTTGAGGTGTTTTACTTGTATTGTTTTTATTTTTTTTCTTTCATTAATTGTAGTATTATATATATGTATATAATAGTCACGACTTTATATATTACAGTCGGAATTAACCCTCACTAA |
| CRP1_CLOX_F2_JP | ACCTTTTATCAATAACCTAAGTCCACATAATGCAGAAATTTATTATTAATTTTGCAAATATCCATTGCGATAAATGTGAAGGAATCATAAGAAACGTTGTGACGGCCAGTGAATTGTAATA |
| CRP1_CLOX_R2_JP | CGACTTTATATATTACAGTTATACTTCGTTTGGTGAAAGCTCACTCTCGGTACTATCATCAACACCAAAATCTTCAGCTTTGAGCAGTGGTTTATACAATTTTCGGAATTAACCCTCACTAA |
| Not1_F_CRP1_ReINT_JP | TTAAATATGTGCGGCCGCGTTACCCAATGCGGTATTCCACCCATTGTCCAATATT |
| Not1_R_CRP1_ReINT_JP | TGTTTTGCGGCCGCTTACTTGTATTGTTTTTATTTTTTTTCTTTCATTAATTGTAG |

^a^ Enzyme restriction sites used for cloning are underlined.
