# Supplementary material for: Host-Imposed Copper Poisoning Impacts Fungal Micronutrient Acquisition during Systemic Candida albicans Infections
Source: PLoS One. 2016 Jun 30;11(6):e0158683. doi: 10.1371/journal.pone.0158683 (PMC4928837; doi:10.1371/journal.pone.0158683)
Supplement: S2 Table — (DOCX) [file pone.0158683.s006.docx]

**S2 Table. Oligonucleotide primers and UPL probes for ploidy verification of *C. albicans* mutant strains.**

| Primer Name | Sequence | UPL Probe^a^ |
| --- | --- | --- |
| JP_ploidy_ACT1_1L | TTATCACTATTGGTAACGAAAGATTCA | 50 |
| JP_ploidy_ACT1_1R | CAGCTTCCAAACCTAAATCAGC |  |
| JP_ploidy_CTR1_1L | GGAATTCTTAAAAAGACACGAAGG | 5 |
| JP_ploidy_CTR1_1R | AGTGGCCGACGTTACCATT |  |
| JP_ploidy_CTR1_2L | TGATGTTGGCTGCTATGACC | 140 |
| JP_ploidy_CTR1_2R | AACACCGGAGCCAATAACC |  |
| JP_ploidy_CRP1_1L | GTTTGATGAAAGTGCATTGACC | 109 |
| JP_ploidy_CRP1_1R | AGATCTGGTGGCCCTCCT |  |
| JP_ploidy_CRP1_2L | TTGAATTTTGGATGGAGTTTAGTG | 3 |
| JP_ploidy_CRP1_2R | CACTGGCCCACACAGGAT |  |

^a^ All primers were designed using the Universal Probe Library Assay Design Centre available on line from Roche Applied Science (http://www.roche-applied-science.com/sis/rtpcr/upl/index.jsp?id=UP030000).
