# Supplementary material for: Host-Imposed Copper Poisoning Impacts Fungal Micronutrient Acquisition during Systemic Candida albicans Infections
Source: PLoS One. 2016 Jun 30;11(6):e0158683. doi: 10.1371/journal.pone.0158683 (PMC4928837; doi:10.1371/journal.pone.0158683)
Supplement: S3 Table — (DOCX) [file pone.0158683.s007.docx]

**S3 Table. Oligonucleotide primers and UPL probes for fungal^a^ and mammalian gene expression analyses.**

| Gene | Primer Name | ORF | Sequence |  | UPL Probe^b^ |
| --- | --- | --- | --- | --- | --- |
| *ACT1* | ACT1_LEFT | orf19.5007 | ACCACCGGTATTGTTTTGGA | | 9 |
|  | ACT1_RIGHT | | AGCGTAAATTGGAACAACGTG | |  |
| *ATO1* | ATO1_LEFT | orf19.6169 | TGGTATCGTTGCTGCTTACG | | 6 |
|  | ATO1_RIGHT | | CAAGGGCATTCTTCAATTGTG | |  |
| *ATO5* | ATO5_LEFT | orf19.6997 | CCTGGTCTTGCACCATATCC | | 18 |
|  | ATO5_RIGHT | | CATACTTGTGGCAACTAATCCAA | |  |
| *ATX1* | ATX1_LEFT | orf19.2369 | CGTAACAATGTCATGCTCAGG | | 92 |
|  | ATX1_RIGHT | | TGATGATACTCCATCTAATCTTTTCAA | | |
| *CDC19* | CDC19_LEFT | orf19.3575 | ACTACCATTGGTGACAAAGATTACC | | 31 |
|  | CDC19_RIGHT | | TCAGTGGTGAAGATCATTTCGT | |  |
| *CRD2* | CRD2_LEFT | orf19.4674 | TTGTGCTGCTGCTCAATGT | | 99 |
|  | CRD2_RIGHT | | AGCTGGTTGTTTACCACATGAA | |  |
| *CRP1* | CRP1_LEFT | orf19.4784 | GTTTGATGAAAGTGCATTGACC | | 109 |
|  | CRP1_RIGHT | | AGATCTGGTGGCCCTCCT | |  |
| *CTR1* | CTR1_LEFT | orf19.3646 | ATTTACAATCACCACCCGTCA | | 18 |
|  | CTR1_RIGHT | | CCGAAACAACTTCTTTTGCAT | |  |
| *CTR2* | CTR2_LEFT | orf19.4720 | TCGTCAAAGCATGGTTCAGT | | 145 |
|  | CTR2_RIGHT | | ATCGTTGAAGCATTGGAAGC | |  |
| *CUP2* | CUP2_LEFT | orf19.5001 | TGATGTCAATAATAATTCTGACGTGAT | | 31 |
|  | CUP2_RIGHT | | GTTGTTGTTATTGCGGTGGTT | |  |
| *DUR1,2* | DUR1,2_LEFT | orf19.780 | CAAATCTCAAACAGTGAATTGGA | | 48 |
|  | DUR1,2_RIGHT | | TCATCAAATTTTTCGTCATCTTG | |  |
| *FOX2* | FOX2_LEFT | orf19.1288 | TGAACATTACTTGAAAGTCCACAAA | | 77 |
|  | FOX2_RIGHT | | CCTTTGGAGTAGTGGAGATTGG | |  |
| *FRE7* | FRE7_LEFT | orf19.6139 | AGGGCCCCTATGGAGAATC | | 78 |
|  | FRE7_RIGHT | | CCGGCAATAAGACCACAAGT | |  |
| *GPA2* | GPA2_LEFT | orf19.1621 | TCCAACTGCAAATGGATCAG | | 77 |
|  | GPA2_RIGHT | | GTGTTTGCGTTGTTTTGGTG | |  |
| *HAP43* | HAP43_LEFT | orf19.681 | GTAACCTGGCACCAAGCAGT | | 58 |
|  | HAP43_RIGHT | | CCCGGTTGGCGATATACTATT | |  |
| *ICL1* | ICL1_LEFT | orf19.6844 | TCCAAAAAGAAGTTGCTGAAATC | | 157 |
|  | ICL1_RIGHT |  | TGGTCTTTCTCCATCTTGGTTC | |  |
| *MAC1* | MAC1_LEFT | orf19.7068 | CAACACTATTTCAACAAGCTCCA | | 65 |
|  | MAC1_RIGHT | | CAAGAACATTCGGAAGACGAA | |  |
| *MLS1* | MLS1_LEFT | orf19.4833 | CTTCCCCATTCCCAAAAAC | | 163 |
|  | MLS1_RIGHT | | TTCTGGGACAGGGCCTAATA | |  |
| *OLE1* | OLE1_LEFT | orf19.5117 | CAACGGTGCTGTATATGCTCA | | 132 |
|  | OLE1_RIGHT | | CTTTAACTACAGCAACTCTCATGGTT | | |
| *PCK1* | PCK1_LEFT | orf19.7514 | TCATTTCATTTCTGGTTACACCTC | | 121 |
|  | PCK1_RIGHT | | GCTTGTGGTTCAGTAACACCTTC | |  |
| *SCO1* | SCO1_LEFT | orf19.7325 | GGAAGCTAACAAGTCCATTGGT | | 119 |
|  | SCO1_RIGHT | | CCTTGGTATCTTGCAACGTAAA | |  |
| *SOD1* | SOD1_LEFT | orf19.2770 | GCTGGTCCTCATTTCAATCC | | 50 |
|  | SOD1_RIGHT | | AATCACCAACATGTCTTTCATCA | |  |
| *SUR7* | SUR7_LEFT | orf19.3414 | TTGTTTTGGCCAAGAATGC | | 121 |
|  | SUR7_RIGHT | | TGATTTGGCACCAATGTGAG | |  |

| *Cp* | CER_Mm_L | MGI:88476 | GCTTATATGGTGGCCCAGAA | 109 |
| --- | --- | --- | --- | --- |
|  | CER_Mm_R |  | CTGGAAAAAGGCCTGCAA | |
| *Ctr1* | CTR1_Mm_L | MGI:1333843 | GGGATCCAGTTCTGAGAGGA | 70 |
|  | CTR1_Mm_R |  | GAAAAAGATGAGATTCAGTGGAAAA | |
| *Gapdh* | GAPDH_Mm_L | MGI:95640 | GGGTTCCTATAAATACGGACTGC | 52 |
|  | GAPDH_Mm_R | | CCATTTTGTCTACGGGACGA | |
| *Atox1* | Mm_ATOX1_L | MGI:1333855 | CCGTCTCCAGAGTCCTCAAC | 52 |
|  | Mm_ATOX1_R | | TGTTGGGCAGGTCAATGTT | |
| *Atpb* | Mm_ATPB_L | MGI:107801 | TGAGAGAGGTCCTATCAAAACCA | 92 |
|  | Mm_ATPB_R | | CACCAGAATCTCCTGCTCAAC | |
| *Ccs* | Mm_CCS_L | MGI:1333783 | TCAAGGGTATGGGCAGTAGC | 32 |
|  | Mm_CCS_R |  | CACAGCCCTCCAGAATGG | |
| *Cox1* | Mm_COX1_L | MGI:102504 | CAGACCGCAACCTAAACACA | 25 |
|  | Mm_COX1_R | | TTCTGGGTGCCCAAAGAAT | |
| *Cox17* | Mm_COX17_L | MGI:1333806 | ATTGAAGCCCACAAGGAGTG | 49 |
|  | Mm_COX17_R |  | TCTTCAAGGATTATTCATTCACAAAG | |
| *Drp1* | Mm_DRP1_L | MGI:1921256 | CTGGATCACGGGACAAGG | 77 |
|  | Mm_DRP1_R |  | GTTGCCTGTTGTTGGTTCCT | |
| *Mfn2* | Mm_MFN2_L | MGI:2442230 | CGAGGCTCTGGATTCACTTC | 21 |
|  | Mm_MFN2_R | | CAACCAGCCAGCTTTATTCC | |
| *Nrf1* | Mm_NRF1_L | MGI:1332235 | TGGAGTCCAAGATGCTAATGG | 100 |
|  | Mm_NRF1_R |  | GCGAGGCTGGTTACCACA | |
| *Sod1* | Mm_SOD1_L | MGI:98351 | CCATCAGTATGGGGACAATACA | 49 |
|  | Mm_SOD1_R | | GGTCTCCAACATGCCTCTCT | |
| *Sod2* | Mm_SOD2_L | MGI:98352 | TGCTCTAATCAGGACCCATTG | 3 |
|  | Mm_SOD2_R | | GTAGTAAGCGTGCTCCCACAC | |
| *Mt1* | MT1_Mm_L | MGI:97171 | CAAGTGCACCTCCTGCAA | 18 |
|  | MT1_Mm_R |  | TTCGTCACATCAGGCACAG | |

^a^ The remaining primers and probes are as given elsewhere [12,30].

^b^ All primers were designed using the Universal Probe Library Assay Design Centre available on line from Roche Applied Science (http://www.roche-applied-science.com/sis/rtpcr/upl/index.jsp?id=UP030000).
